# SEPT9_i1 regulates human breast cancer cell motility through cytoskeletal and RhoA/FAK signaling pathway regulation

**DOI:** 10.1038/s41419-019-1947-9

**Published:** 2019-09-26

**Authors:** Yongqiu Zeng, Yang Cao, Lan Liu, Jiao Zhao, Ting Zhang, Lifan Xiao, Man Jia, Qiang Tian, Hong Yu, Shaokun Chen, Yansen Cai

**Affiliations:** 1 Key Laboratory of Obstetric, Gynecologic, and Pediatric Diseases and Birth Defects, Ministry of Education, Sichuan University, Chengdu, Sichuan China; 2 Department of Medical Cell Biology and Genetics, School of Basic Medical Sciences, Southwest Medical University, Luzhou, Sichuan China; 3 Department of Physiology, School of Basic Medical Sciences, Southwest Medical University, Luzhou, Sichuan China; 4 School of Basic Medical Sciences, Southwest Medical University, Luzhou, Sichuan China

**Keywords:** Metastasis, Focal adhesion, RHO signalling

## Abstract

Increasing cell mobility is the basis of tumor invasion and metastasis, and is therefore a therapeutic target for preventing the spread of many types of cancer. Septins are a family of cytoskeletal proteins with GTPase activity, and play a role in many important cellular functions, including cell migration. SEPT9 isoform 1 protein (SEPT9_i1) has been associated with breast tumor development and the enhancement of cell migration; however, the exact mechanism of how SEPT9_i1 might affect breast cancer progression remains to be elucidated. Here, we report that the expression of SEPT9_i1 positively correlated with paxillin, and both were significantly upregulated in invasive breast cancer tissues of patients with lymph node metastases. Lentivirus-mediated shRNA knockdown of SEPT9 in MCF-7 cells diminished tumor cell migration, focal adhesion (FA) maturation and the expression of β-actin, β-tubulin, Cdc42, RhoA, and Rac, whereas overexpression of SEPT9_i1 in SEPT9-knockdown MCF-7 cells promoted cell migration, FA maturation and relevant protein expression. Furthermore, overexpression of SEPT9_i1 in MCF-7 cells markedly increased FAK/Src/paxillin signaling, at least in part through RhoA/ROCK1 upstream activation. Transcriptome profiling suggested that SEPT9_i1 may directly affect “Focal adhesion” and “Regulation of actin cytoskeleton” signaling mechanisms. Finally, overexpression of SEPT9_i1 markedly enhanced lung metastases in vivo 6 weeks after tumor inoculation. These findings suggest that a mechanism of Septin-9-induced aberrant cancer cell migration is through cytoskeletal regulation and FA modulation, and encourages the use of SEPT9 as novel therapeutic target in the prevention of tumor metastasis.

## Introduction

Distant metastasis of a primary tumor is the major cause of mortality in patients with breast cancer^1^. Some of the key steps in cancer metastasis are the migration and invasion of tumor cells, and these processes require rearrangement of the cytoskeleton and abnormal cell adhesion. The Rho family of GTPases are master regulators of cytoskeleton, and play key roles in assembly and maintenance of cell–cell contacts and cell migration. Given that Rho GTPases are required for cadherin and integrin function, their activity must be regulated to transition between cell–cell adhesion and cell migration^2^. Focal adhesions (FAs) are large macromolecular complexes that link the actin cytoskeleton to the extracellular matrix (ECM) to provide traction, and are critical for cell migration^3,4^. Focal adhesion kinase (FAK), a non-receptor tyrosine kinase, also known as protein tyrosine kinase 2, is a key regulator of growth factor receptor- and integrin-mediated signals, governing fundamental processes in normal and cancer cells through its kinase activity and scaffolding functions^5^. FAK is comprised of multiple protein binding domains, and tyrosine-phosphorylation of several key residues determine protein recruitment, subcellular localization, and catalytic activity of FAK^5^. FAK can regulate cytoskeletal dynamics and cell motility by influencing actin polymerization and FA turnover^6,7^. Studies have shown that increased FAK expression and activity in many cancers is often associated with metastasis and poor clinical outcome, highlighting FAK as a potential determinant of tumor development and cancer progression^8,9^.

Septins are a highly conserved family of GTP binding proteins that have been recently identified as a fourth major component of the cytoskeleton, interacting with microtubules and microfilaments, and linked to a broad spectrum of cellular functions^10^. SEPT9, one of the 13 members of septins, located at the Chr. 17q25 locus and is directly implicated in actin dynamics, angiogenesis, bacterial autophagy, cell motility, cell proliferation, cell shape, cytokinesis, microtubule regulation, vesicle targeting, and exocytosis^11–15^. Alternative splicing results in 18 spliced mRNA variants of SEPT9^16^, and both its expression levels and isoform composition differ among cell types^17^. SEPT9_i1, one of the largest isoforms, has already been involved in ovarian cancer^18^, head and neck cancers^19^, and breast cancer progression^14,20^.

Although it has been reported that there may be an association between SEPT9_i1 and tumor metastasis^14,20^, the mechanism(s) by which SEPT9_i1 directly affects tumor progression remain unknown. Here, we demonstrate that SEPT9_i1 promotes breast cancer cell migration, cytoskeletal rearrangements and FA turnover by targeting Rho/ROCK and FAK signaling in vitro and increases the metastatic potential of breast cancer in vivo.

## Materials and methods

### Cell culture

The human breast cancer cell line MCF-7 was purchased from the Cell Bank of the Chinese Academy of Sciences (Shanghai, China); in addition, HEK293FT cells from Invitrogen/Life Technologies (USA) were used for lentivirus production. MCF-7 cells were incubated at 37 °C and grown in Dulbecco’s Modified Eagle’s Medium (DMEM, Gibco, USA) supplemented with 10% fetal bovine serum (FBS; Biowest, France) and 1% penicillin/streptomycin (HyClone, USA). HEK293FT cells were cultured at 37 °C according to the manufacturer’s instructions. Chemical inhibitors to FAK (10 μM; PF-573228) and ROCK (10 μM; Y-27632) were purchased from Selleck Chemicals (Houston, TX, USA).

### Tissue samples

Paraffin-embedded invasive breast cancer tissues and information from 55 female patients that underwent mammectomy were obtained from the Department of Pathology in the Affiliated Hospital of Southwest Medical University from September 2015 to October 2016. None of the patients had received chemotherapy or radiotherapy before surgery. The study complied with the guidelines of the ethics committee of the Affiliated Hospital of Southwest Medical University. Protein expression of SEPT9 and paxillin in slices were detected by immunohistochemistry as detailed below.

### Immunohistochemical (IHC) staining and data analysis

IHC staining was performed on paraffin sections (4 µm) which were dried for 30 min at 60 °C, dewaxed with xylene, and rehydrated through graded alcohol series. Antigen retrieval was then conducted in citrate buffer (pH 6.0) at 98 °C for 15 min, and endogenous peroxidase was inactivated with 0.3% H_2_O_2_ for 10 min at room temperature. The sections were incubated with rabbit polyclonal anti-SEPT9 (1:150, Abcam, USA) and anti-paxillin antibodies (1:100, Abcam, USA) at 4˚C overnight. After being washed in PBS, the sections were incubated 30 min at room temperature with HRP-conjugated secondary antibody. The immunoperoxidase signal was developed with 3,3’-diaminobenzidine (DAB) solution, and the sections were counterstained with hematoxylin.

Immunohistochemical staining was evaluated at ×400 magnification and scored by two independent pathologists. The staining intensity of tumor cells were categorized as follows: negative (score 0), weak (score 1), moderate (score 2), and strong (score 3). The percentage of positive staining tumor cells was scored as follows: 0 (~5%), 1 (5–25%), 2 (26–50%), 3 (51–75%), and 4 (75–100%). The protein expression of every slice was assessed by using the sum of the staining intensity score and percentage score. Next, this protein expression score was categorized into two groups: 0–3 (low protein expression group) and 4–6 (high protein expression group). The Pearson correlation coefficient was calculated as a measure of correlation between expression of SEPT9 protein and clinicopathological parameters (such as age, tumor size, and the presence of metastases) in breast cancer patients.

### Generation of MCF-7 SEPT9 knockdown and overexpression cell lines

Inverted and self-complementary hairpin structure single stranded nucleotide targeting SEPT9 mRNA and a scrambled negative control oligonucleotide were designed and synthesized by Sangon Biotechnology Company (Shanghai, China). The sequences were as follows:

SEPT9-shRNA, sense: 5′-CCGGAACACCACACACTGTGAGTTTCTCGAG AACTCACAGTGTGTGGTGTTTTTTTG- 3′

scrambled shRNA, sense: 5′-CCGGAACTCCGAACGTGTCACGTTTCTCGA G AACGTGACACGTTCGGAGTTTTTTG-3′

All above sense and antisense oligos were synthesized, annealed, and ligated into a linearized pLKO.1-TRC cloning vector (Addgene, USA). The lentiviral vector was cotransfected with packaging vectors (pLP1, pLP2, and pLP-VSVG) (Invitrogen, USA) into HEK293FT cells. Supernatants containing lentiviruses were harvested 40 h after transfection, and lentiviruses were used to transduce MCF-7 cells. One day after the transduction, puromycin (InvivoGen, USA) was added to the cell culture medium at the final concentration of 1 μg/ml to generate stable MCF-7 cell lines. MCF-7 cells with stably low expression of SEPT9 were diluted and seeded in 96-well plates at 1 cell/well to isolate monoclonal cell lines that were termed as SEPT9-KD1 and SEPT9-KD2, respectively. Other MCF-7 cells were transfected with lentiviral vectors carrying scrambled shRNA (SEPT9-Control). SEPT9-Overexpression cell lines were generated by transduction of SEPT9-KD1 and SEPT9-KD2 cells with lentiviruses harboring full-length SEPT9_i1-C4Flag rescue gene which was under synonymous mutations in cDNA sequences complementary to the above shRNA sequences avoiding its degradation by the shRNA, and a blasticidin resistance gene. The mutant sequences were as follows: CAC AAC GCA TTG CGA ATT C. After 48 h, the transduced cells were selected by growing cells in DMEM growth medium containing 10 μg/ml blasticidin (InvivoGen, USA), and the expression of the rescue gene was verified by western blot analysis and immunofluorescence staining. The cell lines that rescued SEPT9_i1-C4FLAG in SEPT9-KD1 and SEPT9-KD2 cells were termed as SEPT9-Ov1 and SEPT9-Ov2, respectively.

### Wound healing assay

A wound healing (scratch) assay was used to evaluate the ability of SEPT9 to promote migration of MCF-7 cells. Cell lines were seeded into 6-well dishes and grown to 80–90% confluence. A sterile 200 μl pipette tip was used to generate a single wound/scratch across the cell monolayer. The cellular debris from the scraped surface was washed with PBS, and DMEM medium with 2% FBS was added to each well. The images of the cells were taken at 0 h, 24 h, 48 h, and 72 h using phase-contrast microscope. The migration ability of the cells was determined by measuring the width of the monolayer wound for three fields per treatments at 24, 48, and 72 h after scraping, and the migration index was calculated using the following formula, where ‘T’ stands for time (24, 48, or 72 h).

$${\mathrm{Migration}\, \mathrm{index}} = \left( {\frac{{\prime\prime {\it{0}}\prime\prime {\mathrm{h}\,\mathrm{scratch}\,\mathrm{width}} - \prime\prime T\prime\prime {\mathrm{h}\,\mathrm{scratch}\,\mathrm{width}}}}{{0\,{\mathrm{h}\,\mathrm{scratch}\,\mathrm{width}}}}} \right) \times 100$$

The data shown represent the mean and standard deviation of three independent experiments.

### Transwell assays

For migration assays, each cell line was seeded at 5 × 10^4^ in serum-free medium per 8 μm-pore cell culture insert (Corning, USA) that fit into 24-well migration chambers. 500 μl growth medium was added outside of the insert, and plates were incubated at 37 °C for 24 h. The non-migrated cells were scraped from the inner side of the insert, and migrated cells, presenting on the underside of the upper chamber, were fixed with 4% paraformaldehyde (PFA) in PBS and stained with 2% crystal violet-stained. The migrated cells were then photographed under a light microscope at ×100 and were counted in five fields per insert.

### Mice and tumor studies

All animal experiments were performed in accordance with Declaration of Helsinki^21^ in addition to approval granted by the Animal Ethics Committee of Southwest Medical University. Four-week-old female BALB/c nude mice (West China experimental animal center of Sichuan university) were randomized into three groups (*n* = 6): Control (SEPT9-Control cell line injected), Knockdown (SEPT9-KD1 cell line injected), Overexpression (SEPT9- Ov1 cell line injected). For orthotopic injections, the cells (1 × 10^6^ cells per mouse) were injected into the mammary fat pads of mice. Tumors, lungs, and livers were harvested 6 weeks post-orthotopic injection, fixed in 10% neutral buffered formalin (NBF), embedded in paraffin, sectioned, and stained with hematoxylin and eosin (H&E). For tail vein injections, 5 × 10^5^ cells were injected per mouse, and livers and lungs were harvested at 3 weeks post-injection and examined microscopically by H&E staining. Image J was used to segment and count metastases on liver and lung sections per mouse for both spontaneous and experimental metastasis assays.

### Transcriptomic analysis

RNAs from two of the cell lines (SEPT9-KD1 and SEPT9-Ov1) were extracted using Trizol reagent according to manufacturers instructions (Invitrogen, USA); to ensure quality of the RNA samples before sequencing, the NanoDrop 2000 (Thermo Fisher Scientific, USA) and RNA Nano 6000 Assay Kit from the Agilent Bioanalyzer 2100 system (Agilent Technologies, CA, USA) were used to assess the purity, concentration, and integrity of the RNA samples. Transcriptomic analysis was accomplished by Biomarker Technologies (Beijing, China). Differential expression analysis of two conditions/groups was performed using the DESeq R package (1.10.1). The resulting *P* values were adjusted using the Benjamini and Hochberg’s approach for controlling the false discovery rate (FDR). Genes with an adjusted *P*-value < 0.05 found by DESeq were assigned as differentially expressed. Differential expression analysis of two samples was performed using the EBSeq R package. The resulting FDRs were adjusted using the posterior probability of being DE (PPDE). The FDR < 0.05 & |log2 (fold change) | ≥ 1 was set as the threshold for significantly differential expression, and the statistical enrichment of differential expression genes in KEGG pathways was implemented by the KOBAS 3.0 software^22^.

### RNA isolation and real-time PCR

1.2 × 10^6^ cells were seeded in 6-well plates, and total RNA was extracted using Trizol reagent (Invitrogen, USA) one day later. First strand cDNA was synthesized using MLV reverse transcriptase (Invitrogen, USA) and random primers. Quantitative PCR amplification was conducted using AceQ qPCR SYBR Green Master Mix (Vazyme, Nanjin, CHN) with primers specific for human ACTB (sense: 5′-CTCCATCCTGGCCTCGCTGT-3′; antisense: 5′-GCTGTCACCTTCACCGTTCC-3′), CDC42 (sense: 5′-ACGACCGCTGAGTTATCCACAAAC-3′; antisense: 5′-ATACTTGACAGCCTTCAGGTCACG-3′)^23^ and SRC (sense: 5′-CCAGATTGTCAACAACACAGAG-3′; antisense: TCTGACTCCCGTCTGGTGAT-3′). The amplification conditions were: 5 min at 95 °C, 45 cycles of 10 s at 95 °C, 30 s at 53–55 °C, and 40 s at 72 °C, which was performed on a Bio-Rad PCR system (Bio-Rad, CFX96, USA). The relative mRNA expression level was calculated using 2^−ΔΔCt^ method as described previously^24^, with GAPDH as the internal control.

### Immunofluoresence analysis

Cells were fixed with 4% paraformaldehyde for 15 min at room temperature, permeabilized with 0.2% Triton X-100 (EMD Millipore, GER) in PBS for 15 min, and blocked with 5% BSA for 2 h. Then, cells were incubated with primary antibodies against SEPT9 (Abcam, USA), paxillin (Abcam, USA) and Flag ((Santa Cruz, USA), respectively at 4 °C overnight, followed by incubation for 2 h at room temperature with Alexa Fluor® 488 or 568-conjugated secondary antibodies (Invitrogen/Life technologies, USA). Nuclei were stained with DAPI (Sigma), and F-actin was stained with CF568-Phalloidin (Biotium, USA). Images were acquired using the Laser Scanning Confocal Microscope (Olympus, Japan) at ×600 magnification, and the quantification of FA size and number were quantified with ImageJ (NIH, Bethesda, Maryland, USA) with three fields per well and at least five wells per experment.

### Western blotting and COIP analyses

Cells were collected using a cell scraper and suspended in RIPA buffer supplemented with protease inhibitor cocktail and phosphatase inhibitor (Sigma Aldrich, USA). Protein samples were separated by SDS-PAGE and electrically transferred to a PVDF membrane (EMD Millipore, GER). The membrane was then blocked with 5% BSA in TBST (TBS, pH 7.4, 0.2% Tween-20) and incubated at 4 °C overnight with primary antibodies for SEPT9 (Abcam, USA), CDC42 (CST, USA), Rac1/2/3 (CST, USA), t-FAK (CST, USA), p-FAK Y397, Y576/577, Y925 (CST, USA), t-paxillin (Abcam, USA), p-paxillin Y118 (CST, USA), t-Src (CST, USA), p-Src Y419 (CST, USA), p-SrcTyr527 (CST, USA), RhoA (CST, USA), t-ROCK (CST, USA), p-ROCK1 T455 + S456 (CST, USA), β-actin (Santa Cruz, USA), and GAPDH (Santa Cruz, USA). The next day, the membrane was washed 3 times with TBST and incubated with Dylight 680-conjugated secondary antibodies (KPL, USA) in the dark for 2 h.

For COIPs, cells were lysed in cold RIPA lysis buffer supplemented with phosphatase and protease inhibitors (Sigma Aldrich, USA). Lysates were pre-cleared 1 h with binding control protein A/G-agarose beads and incubated overnight with paxillin-Trap agarose beads (Yeasen, USA) at 4 °C. Beads were washed in IP lysis buffer, and bound protein was eluted. All membrane images were acquired with the Li-Cor Odyssey Clx Infrared Imaging System (LI-COR Biotechnology, Lincoln, NE, USA) and densitometry analysis of bands was performed using ImageJ software.

### Statistical analysis

Each experiment was performed at least 3 times. Values are shown as means ± SEM. Significance was determined by unpaired Student’s *t*-test for two-group comparisons and one-way ANOVA for comparisons of more than two groups. Pearson’s correlation analysis was used to analyze the correlation between two indices. *P* < 0.05 was considered statistically significant.

## Results

### A significant positive correlation between SEPT9 and nodal metastasis and the expression of paxillin in breast cancer tissues

**Fig. 1:**
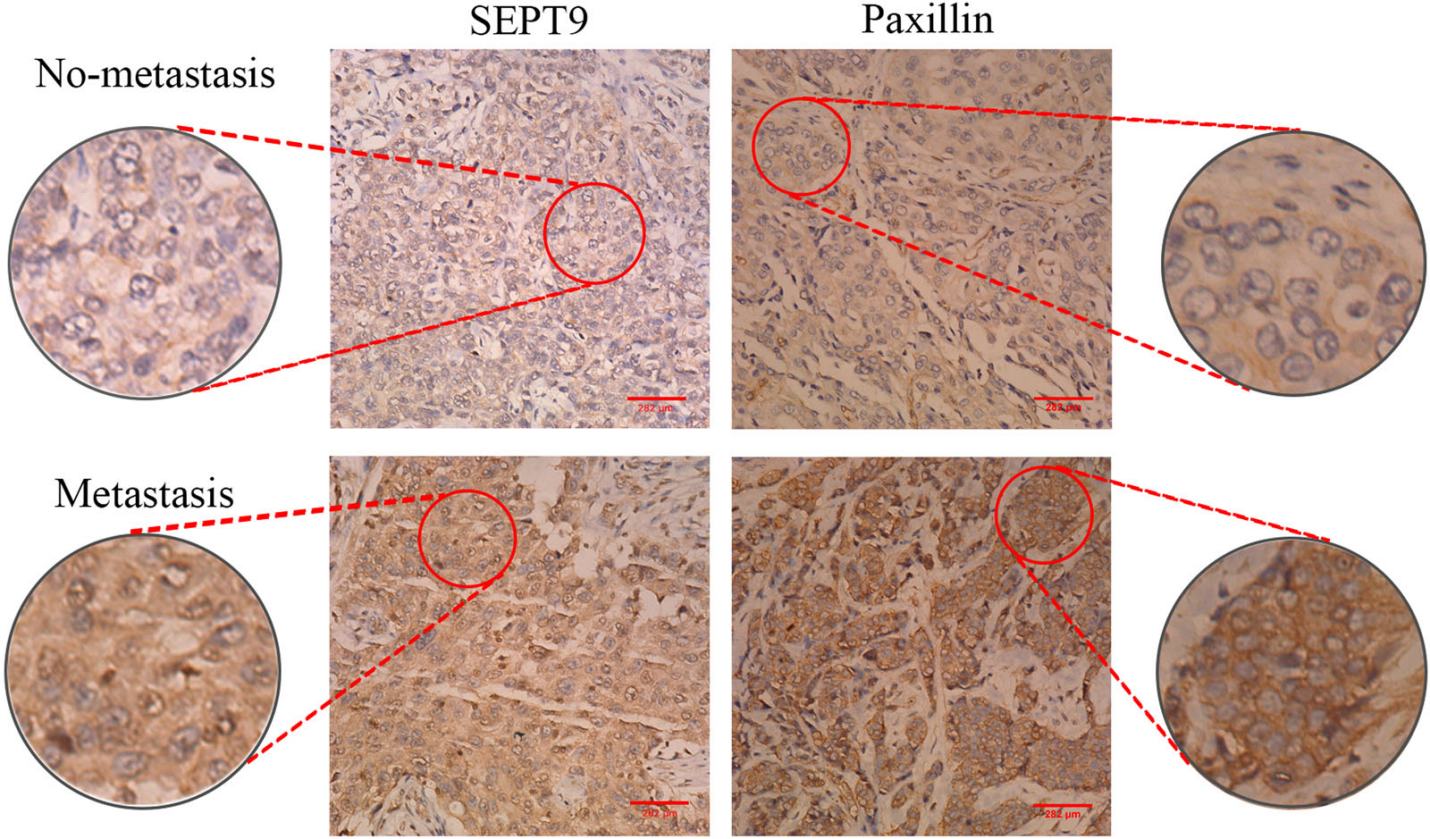
Representative images of SEPT9 and paxillin protein expression in human breast cancer tissues with lymph node metastasis (lower panels) and no-metastasis (upper panels) were detected via immunohistochemical staining (×400)

In this study, we first examined the correlation of SEPT9 with clinical characteristics and paxillin in breast cancer samples. Our results showed that SEPT9 and paxillin were overexpressed in 74.5% (41/55) and 54.5% (30/55) of the primary breast cancer clinical samples, respectively (Table 1). The expression levels of SEPT9 and paxillin were significantly increased in the lymph node metastasis group compared to the non-lymph node metastasis group (Fig. 1), and SEPT9 expression was positively correlated with lymph node metastasis (*γ* = 0.367, *P* = 0.006). Moreover, there was a positive correlation between SEPT9 and paxillin in the cancer tissues (*γ* = 0.665, *P* = 0.000; Table 1).

**Table 1:** Correlation between expression of SEPT9 protein and clinicopathological parameters in breast cancer patients (*n* = 55)

Variables	SEPT9-Negative		SEPT9-Positive		*γ*, *P*
	No. of patients (*n* = 14)	%	No. of patients (*n* = 41)	%	
Age (years)					0.011, 0.936
≤45	5	36%	18	44%	
>45	9	64%	23	56%	
Tumor size (cm)					0.191, 0.163
≤2	5	36%	16	39%	
>2	9	64%	25	61%	
Metastatic lymph nodes					0.367, 0.006**
Negative	10	71%	19	46%	
Positive	4	29%	22	54%	
Paxillin					0.665, 0.000**
Negative	11	79%	11	27%	
Positive	3	21%	30	73%	

**p < 0.01

### SEPT9_i1 expression influences the migration of MCF-7 cells in vitro

**Fig. 2:**
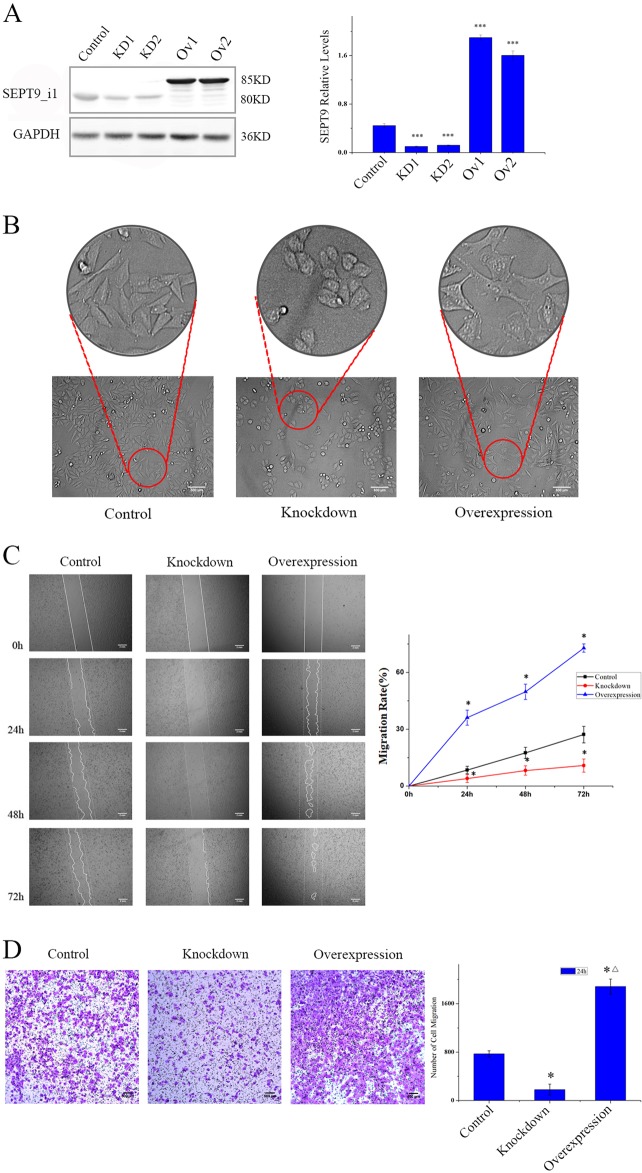
Effects of SEPT9_i1 on cell morphology, motility and migration in MCF-7 cell lines. **a** Western blot for SEPT9_i1 in control, two SEPT9-Knockdown MCF-7 cell lines and two SEPT9-Overexpression cell lines (***p < 0.001). **b** Phase-contrast microscope images of control, SEPT9-knockdown and SEPT9-Overexpression cells with altered cell morphology (×200). **c** The motility of cells was measured by a wound healing assay (×40) (*P < 0.05, compared with control group). **d** Transwell migration assays demonstrating the migration potential of control, SEPT9-Knockdown and SEPT9- Overexpression cells (×100) (*p < 0.05, significant difference versus control; △*p* < 0.05, significant difference versus knockdown)

To examine how SEPT9_i1 functions in the motility of MCF-7 cells in vitro, we inhibited the expression of SEPT9 in MCF-7, which is a low metastatic breast cancer cell line. The two clones stably expressing shSEPT9 (KD1 and KD2) exhibited over 70% protein reduction, while the SEPT9_i1 overexpressed cell lines (Ov1 and Ov2) showed more than tripled compare with scrambled control group (Fig. 2a). Unlike control cells, SEPT9 knockdown MCF-7 cells did not spread out or form protrusions, and SEPT9_i1 overexpression caused markedly altered cell morphology with increased formation of lamellipodia and filopodia, which corroborates previous observations^25^ (Fig. 2b). Furthermore, SEPT9-Knockdown cells exhibited decreased motility and migration, while SEPT9_i1overexpressed cells exhibited significantly increased motility and migration compared with the scrambled control cells (Fig. 2c, d). These data confirmed that SEPT9_i1 is important for the motility and migration of MCF-7 cells in vitro (Knockdown represents the data from KD1 and Overexpression represents Ov1in these figures, the same below. The data from KD2 and Ov2 showed in Supplementary Fig. 1)

### Overexpression of SEPT9_i1 promotes metastasis of tumor cells to the lungs

**Fig. 3:**
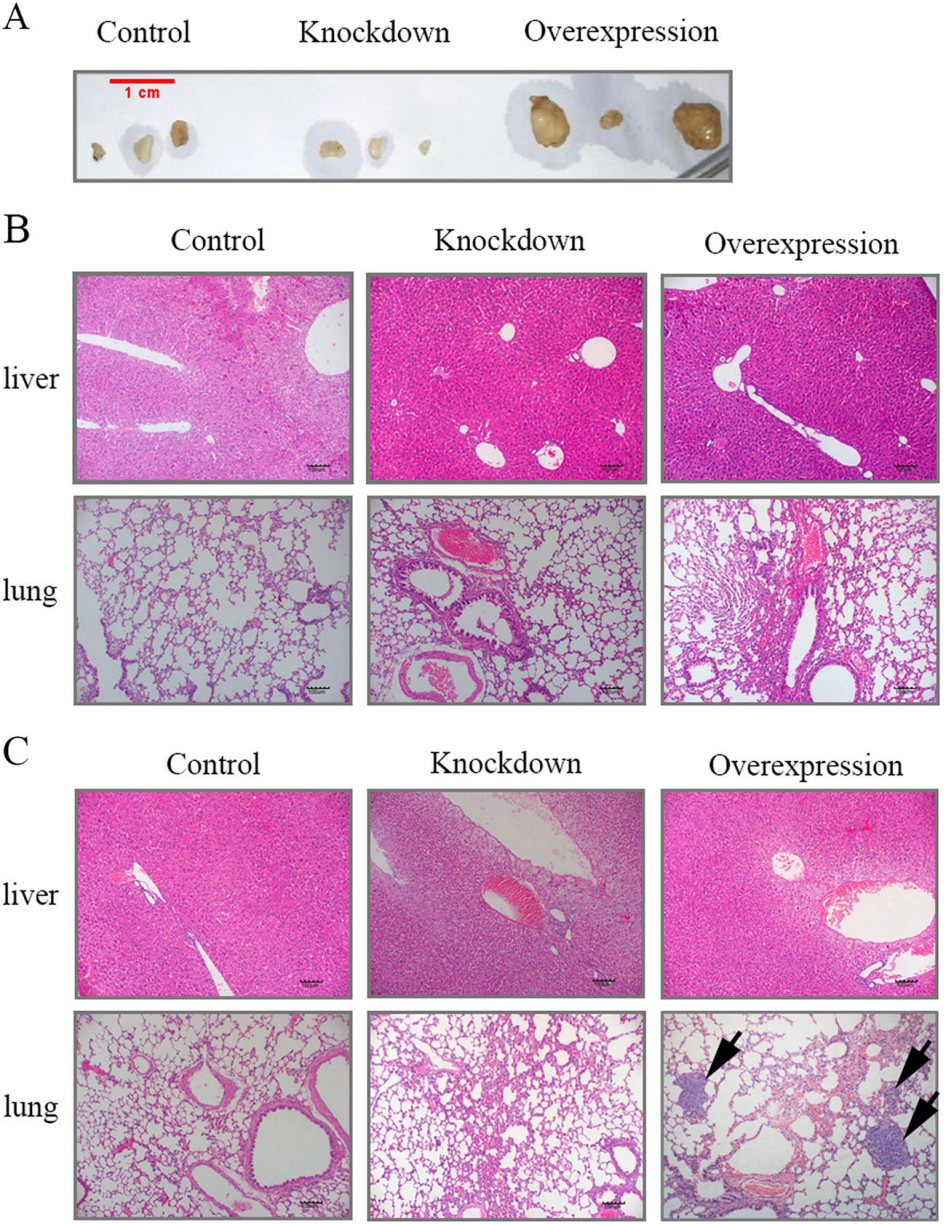
Overexpression of SEPT9_i1 promotes lung metastases after tail vein injection. **a** 1 × 10^6^ cells per group were injected into the mammary fat pads of nude mice. Shown are representative tumors that formed 6 weeks post-injection. **b** Representative H&E-stained sections of lungs and livers of mice with control, knockdown and overexpression group tumors 6 weeks post-orthotopic injection. **c** Representative H&E-stained sections of lungs and livers of mice with control, knockdown and overexpression group tumors 3 weeks after tail vein injection

To investigate the effects of SEPT9_i1 expression on breast tumor metastasis in vivo, we injected the engineered MCF-7 cells either into mammary fat pads or the general circulation (via tail vein injection) of BALB/c female nude mice. Palpable mammary tumors were observed within 6 weeks when cells were inoculated into mammary fat pads (Fig. 3a), but no obvious metastases were found in lung or liver sections in mice injected with any of the cell lines (Fig. 3b). However, 3 weeks after tail vein injections, many macro-metastases were evident in lung sections from mice with SEPT9-Overexpression tumors but not mice with SEPT9-Knockdown and scrambled control tumors (Fig. 3c). These data indicate that SEPT9_i1 might promote tumor cell survival and motility in the circulation or metastatic outgrowth at secondary sites, but do not assist escaping from the primary tumor.

### RNA-seq highlights SEPT9-induced transcriptional changes in FA and actin cytoskeleton regulation

**Fig. 4:**
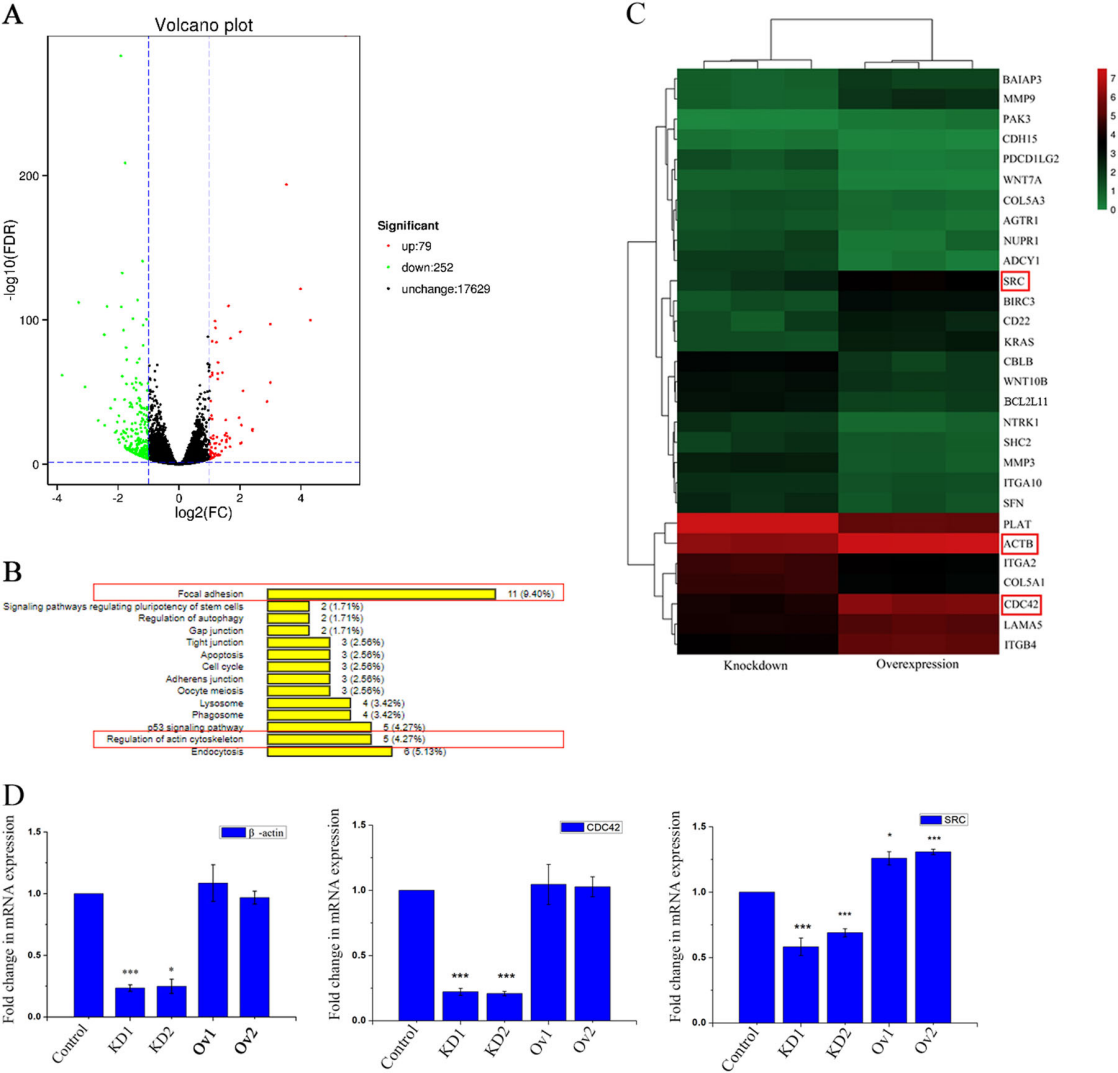
Bioinformatic analysis of DEGs between SEPT9-KD1 and SEPT9-Ov1 cell lines. **a** Volcano plots for all the genes comparing SEPT9-KD1 and SEPT9- Ov1. The red and green dots indicated up- and down-regulated DEGs with FDR < 0.05, while black dots indicated those without statistical significance. **b** KEGG pathway terms enriched by DEGs between SEPT9-KD1 and SEPT9- Ov1 cell lines, here we listed reference canonical pathways (all *p* < 0.05) belonging to “Cellular Process” and “Environmental Information Processing”. **c** Heatmap plot of some related differentially expressed genes across all samples. Columns represent genes and rows represent samples. **d** β-actin, CDC42 and SRC mRNA expression were analyzed by real-time RT-PCR (***p < 0.001, *p < 0.05)

RNA-seq of two cell lines (SEPT9-KD1 and SEPT9-Ov1) was performed. Using FC (Fold change) > 2 and FDR < 0.05 as the statistical cutoffs, we identified 331 differentially expressed genes (DEGs) between SEPT9-KD1 and SEPT9-Ov1 cell lines. Among these genes, 79 genes were upregulated with SEPT9_i1 overexpression, and 252 genes were down-regulated (Fig. 4a). The cellular processes affected by SEPT9 overexpression were evaluated by mapping the DEGs to reference canonical pathways in the KEGG (Kyoto Encyclopedia of Genes and Genomes) database. Eleven DEGs were categorized in the “FA” pathway and five DEGs were in the “Regulation of actin cytoskeleton” pathway (Fig. 4b). Changes in these two pathways are commonly observed during cell migration and changes in cell motility^4,6^, so these categorical annotations provided evidence of a role for SEPT9 in established signaling mechanisms. The heatmap for these and some cancer related DEGs was shown in Fig. 4c. β-actin, CDC42 and SRC mRNA were proved to conform to the changes of DEGs by real-time RT-PCR (Fig. 4d).

### SEPT9_i1 influences the expressions of GTPases and interacts with stress fibers, microtubules

**Fig. 5:**
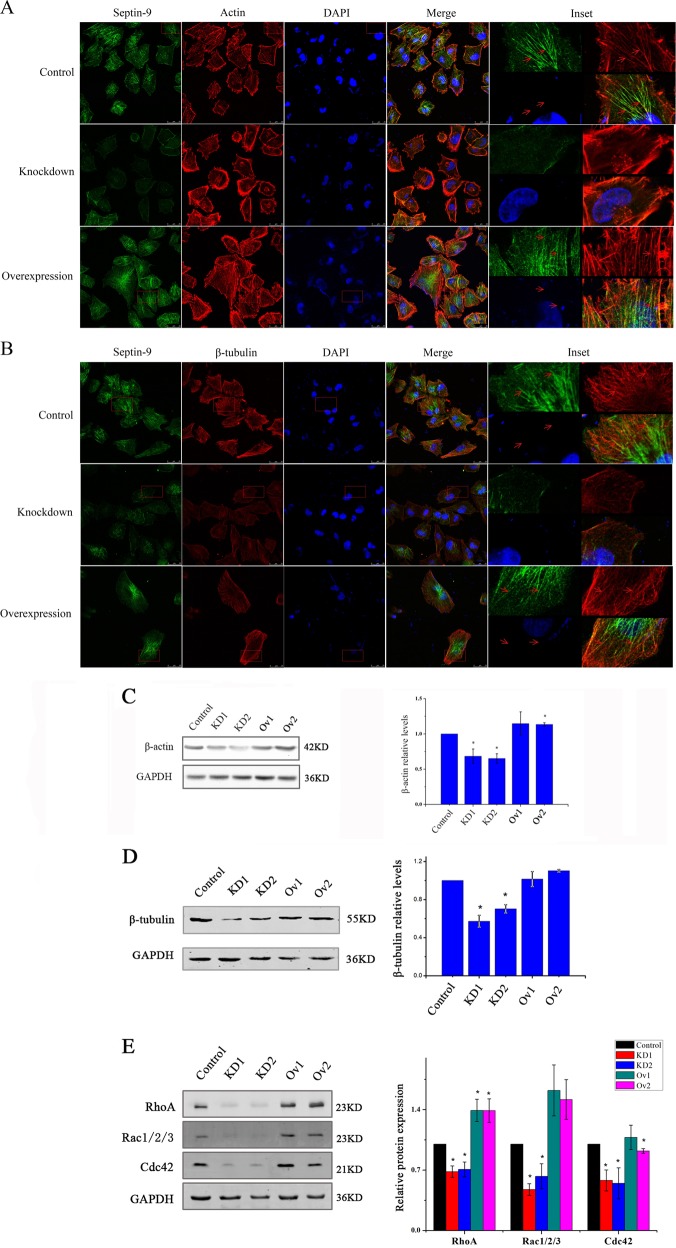
Septin-9 filaments colocalize with F-actin and microtubules, regulate the expression of β-actin and β-tubulin. **a** Confocal images of septin-9 (green) and actin stress fibers (phalloidin; red) in control, knockdown and overexpression group cells. 3D reconstruction reveals that septin-9 filaments colocalize with F-actin stress fibers. **b** Confocal images of septin-9 (green) and microtubules (red) in control, knockdown and overexpression group cells. It reveals that septin-9 filaments colocalize with microtubules. **c**, **d** β-actin and β-tubulin protein expression were analyzed by western blot (*p < 0.05). **e** Cdc42, Rac and RhoA protein expression were analyzed by western blot

**C**onfocal microscopy and 3D image reconstruction were used to more precisely examine the effects of SEPT9_i1 expression on the cytoskeletal network of MCF-7 cells. The actin meshwork was thin and devoid of organized stress fibers in the central region of SEPT9-Knockdown cells rather than in control and SEPT9-Overexpression cells (Fig. 5a). Similarly, a network of microtubules also displayed thin and disorder in SEPT9-Knockdown cells (Fig. 5b). In addition, septin-9 appeared to be partially co-localized with stress fibers and microtubules in SEPT9-Ov and scrambled control cells obviously (Fig. 5a, b). Western blotting showed that gene and protein expression of β-actin are down-regulated in SEPT9 knockdown cells as well as β-tubulin protein (Fig. 5c, d). Additionally, GTPases Cdc42, Rac and RhoA, which are critical for actin network organization, were all decreased in SEPT9-KD cells compared to control, and increased in SEPT9-Ov cells (Fig. 5e). Taken together, the possible interaction of septin-9 with F-actin and microtubules, combined with the altered expression of β-actin and β-tubulin, Cdc42, Rac, RhoA in SEPT9 engineered cells lends insight into mechanisms by which SEPT9_i1 may contribute to organization of cytoskeleton in breast cancer cells.

### SEPT9_i1 interacts with paxillin and promotes maturation of FAs

**Fig. 6:**
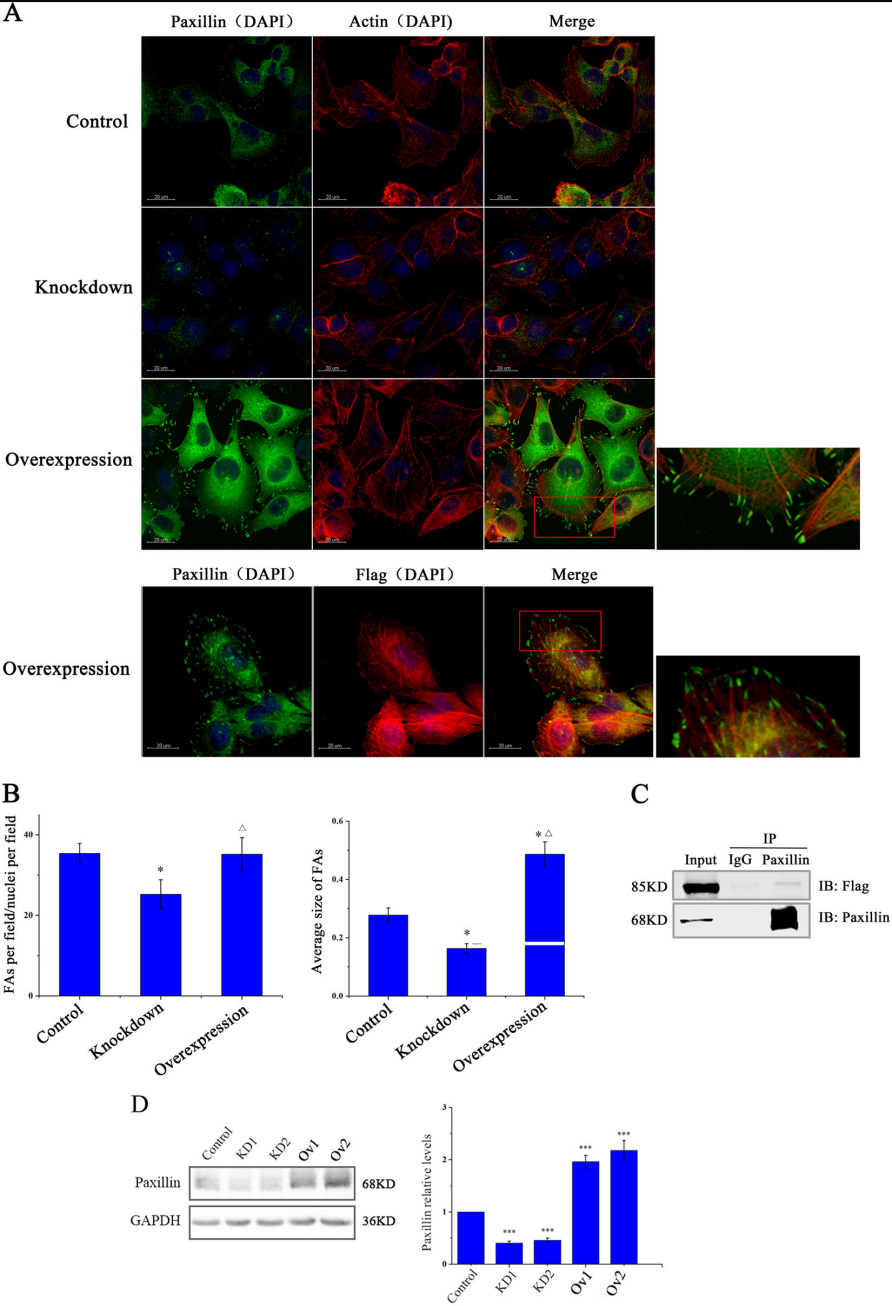
The effect of SEPT9 on the number and size of focal adhesions in breast cancer cells and the interaction between SEPT9_i1 and paxillin. **a** IF of FA proteins paxillin in control, SEPT9-KD and SEPT9-Ov cells. IF of SEPT9_i1 (red) and paxillin (green) in SEPT9-Ov cells. **b** Quantification of FAs and average FA size per nucleus (*p < 0.05, vs control group; △*p* < 0.05, vs Knockdown group). **c** Interaction between SEPT9_i1 and paxillin by COIP. **d** Western blot confirming the inhibition in the expression of paxillin in knockdown group and overexpression of SEPT9_i1 promoting the expression of paxillin (*p < 0.05, ***p < 0.001)

Analysis of paxillin IF in MCF7 cells revealed that the size and number of FAs in SEPT9 knockdown cells were reduced compared to the SEPT9-Ov line, which resulted in large, membrane-associated FAs (Fig. 6a, b). We also observed that part of SEPT9_i1 fibers closer to the cell edge directly connected to FAs same as the stress fibers (Fig. 6a). COIP assay discovered that SEPT9_i1 interacted with paxillin (Fig. 6c), and western blotting assays revealed that paxillin protein were decreased in SEPT9-KD cells, and SEPT9-Ov increased expression compared with scrambled control cells (Fig. 6d). Combined with observations of SEPT9_i1-induced changes in actin and microtubules cytoskeleton, these data indicate that SEPT9_i1-mediated FA maturation may be related to the organization of the cytoskeleton, and expression and localization of paxillin. (Supplementary Fig. 2)

### Overexpression of SEPT9 in MCF-7 cells activates RhoA/ROCK1 and FAK/Src/paxillin signaling, and promotes cell migration

**Fig. 7:**
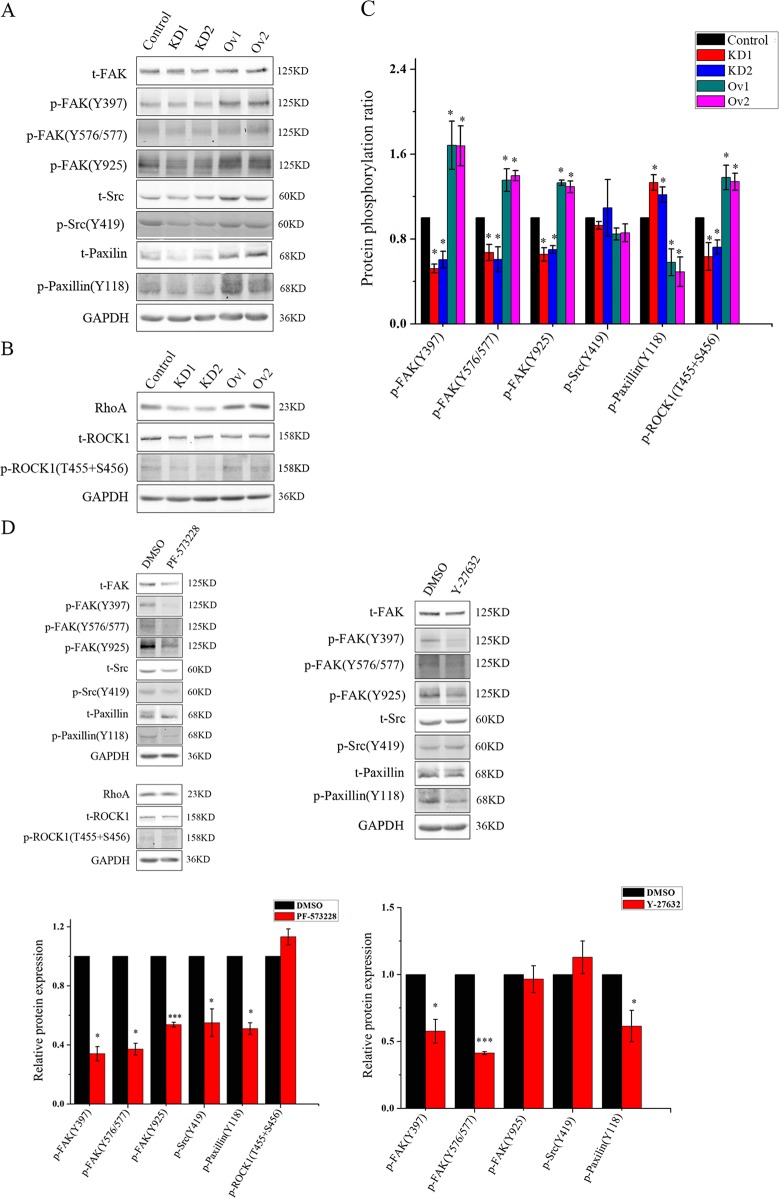
RhoA-ROCK1 and FAK-Src-paxillin signaling are important for the migration of MCF-7 cells. **a**–**c** Cell lysates from Control, two cell lines with SEPT9 knockdown and two cell lines with SEPT9_i1 overexpressed were immunoblotted for total FAK, Src, paxillin, RhoA, ROCK1, their phosphorylated protein levels and phosphorylation ratio. **d** Effects of PF-573228 and Y-27632 on the expression of FAK-Src-paxillin and RhoA-ROCK1 signaling related proteins (*p < 0.05, ***p < 0.001)

FAK is a non-receptor tyrosine kinase linked to FA dynamics^26^ and the FAK/Src/paxillin signaling cascade is known to be involved in several cellular processes, including migration and adhesion^27^. In addition, RhoA/ROCK1 signaling plays a key role in stabilizing actin filaments and regulation of the actin cytoskeleton^28^. Given our observations of an association between SEPT9 and actin, we investigated proteins in the FAK/Src/paxillin and RhoA/ROCK1 signaling pathways, as well as their phosphrorylation. We found that SEPT9 knockdown abolished activation of the FAK-Src-paxillin pathway, where p-FAK (Y397, Y576/577 and Y925), p-Src (Y416), p-paxillin (Y118) (Fig. 7a), and p-ROCK1 (T455 + S456) (Fig. 7b) were all inhibited compared to control cells (Supplementary Fig. 3). Compared to the total proteins, phosphorylation of FAK and ROCK1 were significantly higher in SEPT9_i1 overexpression cells than in control or SEPT9-Knockdown cells (Fig. 7c). These results suggest that SEPT9_i1 can mediate activation of the FAK/Src/paxillin and RhoA/ROCK1 pathways, which could affect cell migration.

To shed light on the upstream and downstream relationship between FAK/Src/paxillin and RhoA/ROCK1 signaling in SEPT9-mediated MCF-7 cell migration, a selective FAK inhibitor, PF-573228, and a ROCK inhibitor, Y-27632, were used in SEPT9 overexpression MCF-7 cells. The transwell and wound healing assays revealed that the migration and motility of the cells were both inhibited after treatment with the inhibitors, and both the number and size of FAs in these cells were reduced (Supplementary Fig. 4). Additionally, the FAK/Src/paxillin signaling pathway was inactivated by Y-27632, while the activity of RhoA-ROCK1 signaling was not affected after treatment with the FAK inhibitor PF-573228 (Fig. 7d). These data indicated that RhoA-ROCK1 might play a role upstream of FAK-Src-paxillin signaling.

## Discussion

SEPT9_i1, a product of SEPT9 transcript variant 1 (*SEPT9_v1*) was shown to be frequently increased in breast tumors, and has already been involved in these tumors progression^29^. In this study, we used shRNA lentivirus vector to knockdown the expression of SEPT9 isoforms in MCF-7 cells significantly including SEPT9_i1, and the reduced cell motility, cell migration and the formation of invadopodia were detected. These effects were recovered when rescued the expression of foreign SEPT9-i1 specially, and these results agree fairly well with the findings in human prostate cancer cells^30^ and immortalized murine embryonic fibroblasts in mice homozygous deletion of SEPT9^31^. Importantly, although SEPT9_i1 overexpression cells were unable to metastasize successfully from an orthotopic site, they were able to colonize the lung when injected directly into the circulation, which indicated that SEPT9_i1 can promote the survival and migration in the circulation and outgrowth in the lung.

Cell migration requires reorganization of actin and microtubules, which plays a critical role in cancer metastasis^32^. SEPT9 has been shown to be involved in the reconstruction process by interacting with actin filaments directly and indirectly^33,34^. A recent study demonstrated that septin-9 binds to F-actin in a highly polymorphic fashion and protects actin filaments from depolymerization by cofilin and myosin, hence, SEPT9 could maintain the integrity of growing and contracting actin filaments^34^. However, actin filaments were on average slightly thinner in *SEPT*9^del/del^ cells compared to *SEPT9*^cond/cond^ cells^31^. This study showed that septin-9 partially co-localized with stress fibers; when SEPT9 was knocked down, actin fibers were reduced and positioned to the cortical edge of the cell, while SEPT9_i1 overexpression substantially increased stress fibers. Based on these observations, we hypothesized that if the protective effect of SEPT9_i1 fibers on F-actin was reduced by knockdown of SEPT9, then the depolymerization of F-actin would be enhanced. However, when the protective effect was recovered with overexpression of SEPT9, the depolymerization of F-actin was reduced and stability of F-actin was enhanced. In mammalian cells, septin depletion affects MT organization, dynamics, and post-translational modifications^35,36^. SEPT9_i1 has already been proposed to participate in the resistance to MT-disrupting agents^37^, and cell resistance to paclitaxel^38^. It also is the only septin in which specific repeated motifs might allow MT binding and bundling^39^. We observed the coalignment between SEPT9_i1 and microtubule, and also showed microtubule organization was altered after SEPT9 knockdown, and recovered when SEPT9_i1 overexpressed. Interestingly, the expression of β-actin and β-tubulin was decreased in SEPT9-knockdown cells; whether this is a direct or indirect association is unclear and requires further mechanistic studies.

Cell migration also consists of well-defined steps that include extension of the leading edge and the formation of immature FAs; FA maturation and cell body translocation; and the FA disassembly and rear retraction^40^. During this cycle, FA dynamics and cytoskeletal organization conjoin to drive this coordinated process^4^. The actin cytoskeleton assembles into branched networks or bundles to generate mechanical force for critical cellular processes such as establishment of polarity, adhesion, and migration^41^. FAs undergo a maturation process, which is known to be induced by biochemical or physical stimuli, to vary FA size, quantity, distribution, dynamics, and composition^40^. This process is regulated by tension forces from actin stress fibers^40,42,43^. Microtubules are also involved in the organization and turnover of FAs^44,45^. Microtubules play an important role in the delivery of proteins to subcellular locations including the FAs and they are critical in paxillin assembly in cell periphery and lamellipodia^46^. Recent research indicates that in the leading edge of motile renal epithelia, a novel network of septin filaments promotes cell motility by reinforcing the organization of the lamellar stress fiber network and the stability of nascent FAs^33^. Our work adds to a growing body of work linking SEPT9, F-actin, microtubules and FAs to tumor cell motility, migration, and metastasis, especially the link between SEPT9_i1 and paxillin was first suggested by IF and COIP analysis. Whether the interaction between SEPT9_i1 and paxillin can influence the stability of paxillin protein, and then caused the difference expression of paxillin protein similar to Hif-1α^47^, We will make further research. Furthermore, we had revealed that SEPT9 knockdown might influence the cargo transport through the microtubules in previous research (Supplementary Fig. 2), and in this research, SEPT9 knockdown decreased the expression of β-tubulin, microtubules organization, as well as the number and size of FAs. Hence, SEPT9_i1 might influence on the turnover of FAs partly through altered the microtubule delivery function of paxillin. Not only that, we observed that in contrast to SEPT9-knockdown cells, the FAs in SEPT9_i1 overexpression cells were larger and closer to the edge of the cells, which implies SEPT9_i1 might promote cell migration through facilitating actin and microtubule cytoskeleton reorganization, the stabilization of cytoskeleton, and advancing FA maturation.

FAK is ubiquitously expressed in many different cells and tissues, and functions as both a protein tyrosine kinase and scaffold, mediating and regulating specific signals initiated at the sites of integrin clusters of FAs^48^. Several types of signaling events can initiate FAK activation, which leads to a number of cellular processes, including attachment, migration, invasion, proliferation, and survival^49^. The well-documented example of FAK function involves clustering of the integrins and the subsequent co-clustering of proteins such as talin and paxillin with the cytoplasmic tail of integrins^50,51^. FAK contains multiple tyrosine and serine phosphorylation sites that can induce conformational changes^52^, including Y397, Y576/Y577, and Y925. The FAK–Src complex phosphorylates and/or recruits several downstream FA proteins, including paxillin, p130Cas, etc., initiating specific cellular signaling pathways and responses^53^. Deregulated FAK/Src signaling occurs in a variety of tumors^8,50,54^. FAK/Src-dependent phosphorylation of paxillin Y31/118 exposes a vinculin binding site^55^ and recruits other adhesion molecules. Increased adhesion forces then transmit stronger mechanical signals to promote focal adhesion complex turnover in the process of cell migration^7,56–58^. In this study, our novel findings suggest a relationship between SEPT9 and activation of the FAK/Src/paxillin pathway. FAK-Y397, -Y576/577, -Y925, and Src-Y416 were all found to be phosphorylated in SEPT9_i1 overexpression cells, which implied the activation of FAK-Src-paxillin signaling. However, the results from transcriptomic analysis showed that *ITGB4* is one of DEGs between SEPT9-KD1 and SEPT9-Ov1 cell lines, which suggested that the activation of FAK-Src-paxillin signaling might partly result from the increased expression of ITGB4 as the previous researches reported^59,60^. Notably, previous studies have demonstrated that the maturation of FAs is force dependent^55,61^, and the phosphorylation of paxillin plays a central role in this process^58^. In line with this, we found that there was a reduction in the phosphopaxillin-to-paxillin ratio when SEPT9_i1 was overexpressed (Fig. 7c), which is consistent with evidence that the relative level of phosphopaxillin is negatively regulated by force^61,62^.

The ability of Rho GTPase family members to regulate cytoskeletal dynamics, cell adhesion, and cell migration points to a central role in cancer cell invasion and metastasis. Of this family, RhoA, Rac, and Cdc42 typically control the actin and microtubule cytoskeleton organization, cell motility and cell adhersion^32^. Constitutively activated (GTPase deficient) mutants of Rho and Rac were found to induce the assembly of contractile actin and myosin filaments (stress fibers) and actin-rich surface protrusions (lamellipodia). Cdc42 has also been shown to promote the formation of actin-rich, finger-like membrane extensions (filopodia). These specialized actin structures are believed to provide the driving force for cell migration^25^. In this research, the protein expressions of RhoA, Rac and Cdc42 were all reduced in SEPT9-Knockdown cells and restored when SEPT9_i1 was overexpressed, and so was *CDC42* mRNA (Fig. 4). Corresponding changes in actin and microtubules cytoskeleton, microtubule dynamics and cell morphology were also seen. These GTPase proteins have been known to reorganize cytoskeletons and to regulate cell migration via activation of effector proteins such as ROCK^63–66^. As a major signaling node for the interfaces of cell–cell adhesion, RhoA signaling is triggered when mechanical forces are applied to a variety of cell adhesion molecules^67^, and it has been a main focal point in studies of cellular responses to mechanical forces^68^. Activated RhoA can stimulate actin polymerization^69^ and also stabilize F-actin by activating ROCK^70^. It is well established that the Rho/ROCK signaling pathway is associated with cancer invasion and metastasis, such as breast cancer^71,72^, ovarian cancer^63^, colon cancer^73^, and gastric cancer^65^.

Previous studies have demonstrated that there is crossover and interaction between RhoA/ROCK1 and FAK/Src/paxillin signaling in promoting cell migration, and they may share upstream or downstream effector molecules and signaling processes^74–76^. Here, we also demonstrated that RhoA/ROCK1 signaling may play an important role in activation of FAK/Src/paxillin signaling, which was similar to the molecular mechanism of the hypoxia-induced breast cancer cell migration^76^. Earlier reports had identified that SA-RhoGEF (septin-associated RhoGEF) and Rhotekin can binds with SEPT9 and altered endogenous septin filament structures. They are novel regulators organizing mammalian septin structures and provide a link between septins and Rho signaling^77,78^. These results remind us SEPT9_i1-induced activation of RhoA/ROCK1 might be through interaction with these Rho regulators.

In summary, our results provide in vitro and in vivo evidence for the contribution of SEPT9_i1 to the motility of breast cancer cells. These studies showed that SEPT9 overexpression increased cell migration and motility through reorganization of cytoskeleton. Activation of the RhoA/ROCK1 and FAK/Src/paxillin pathways promoted FA maturation, which was consistent with KEGG pathway analysis. These results provide a basis for further investigation into targeting SEPT9-related signaling in order to effectively decrease the metastatic potential of tumor cells in breast and other carcinomas.

## Supplementary information

Supplementary Fig 1

Supplementary Fig 2

Supplementary Fig 3

Supplementary Fig 4
