# Supplementary figures and images for: SEPT9_i1 regulates human breast cancer cell motility through cytoskeletal and RhoA/FAK signaling pathway regulation
Source: Cell Death Dis. 2019 Sep 26;10(10):720. doi: 10.1038/s41419-019-1947-9 (PMC6763430; doi:10.1038/s41419-019-1947-9)

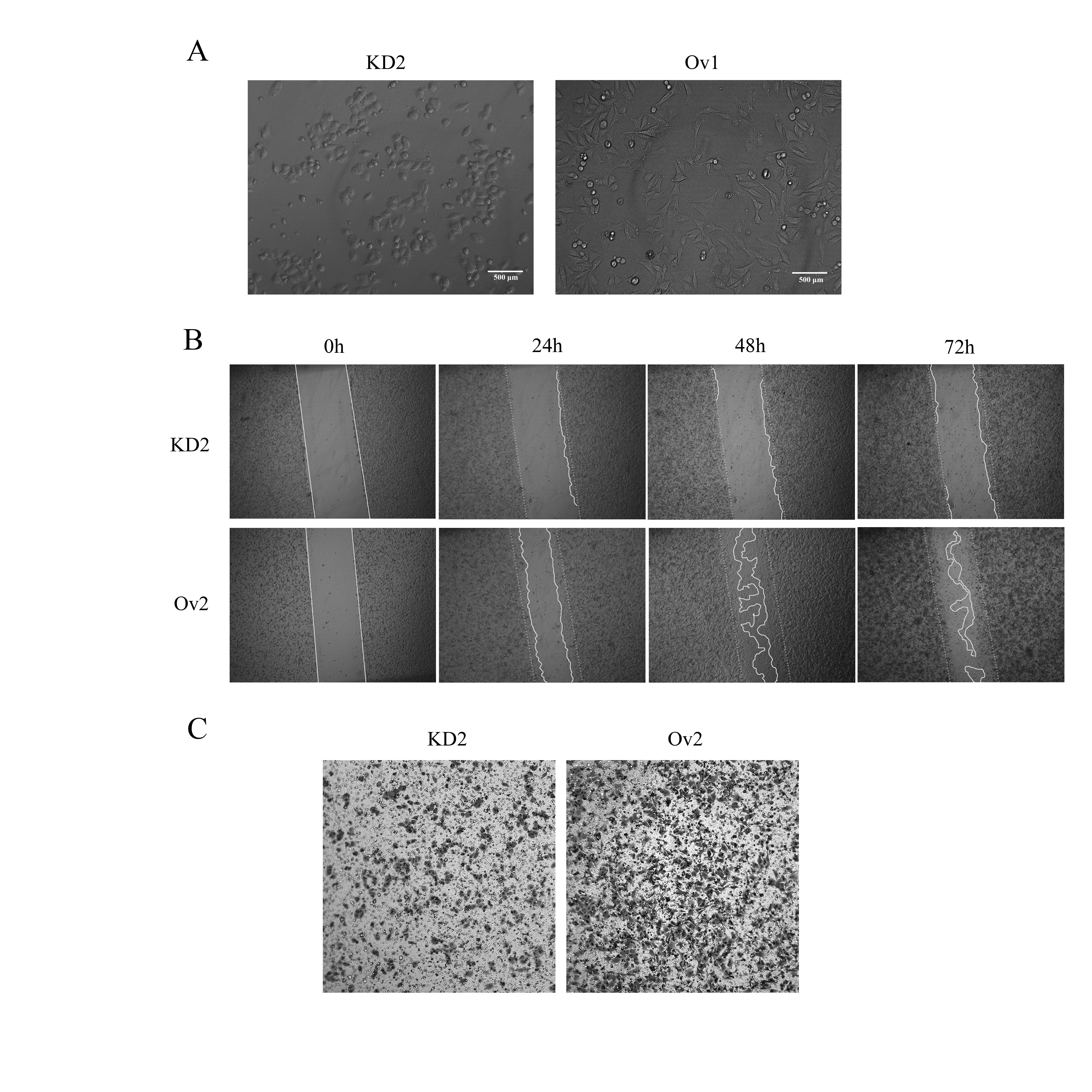

Supplement: Supplementary file 1 — Supplementary Fig 1 [file 41419_2019_1947_MOESM1_ESM.jpg]

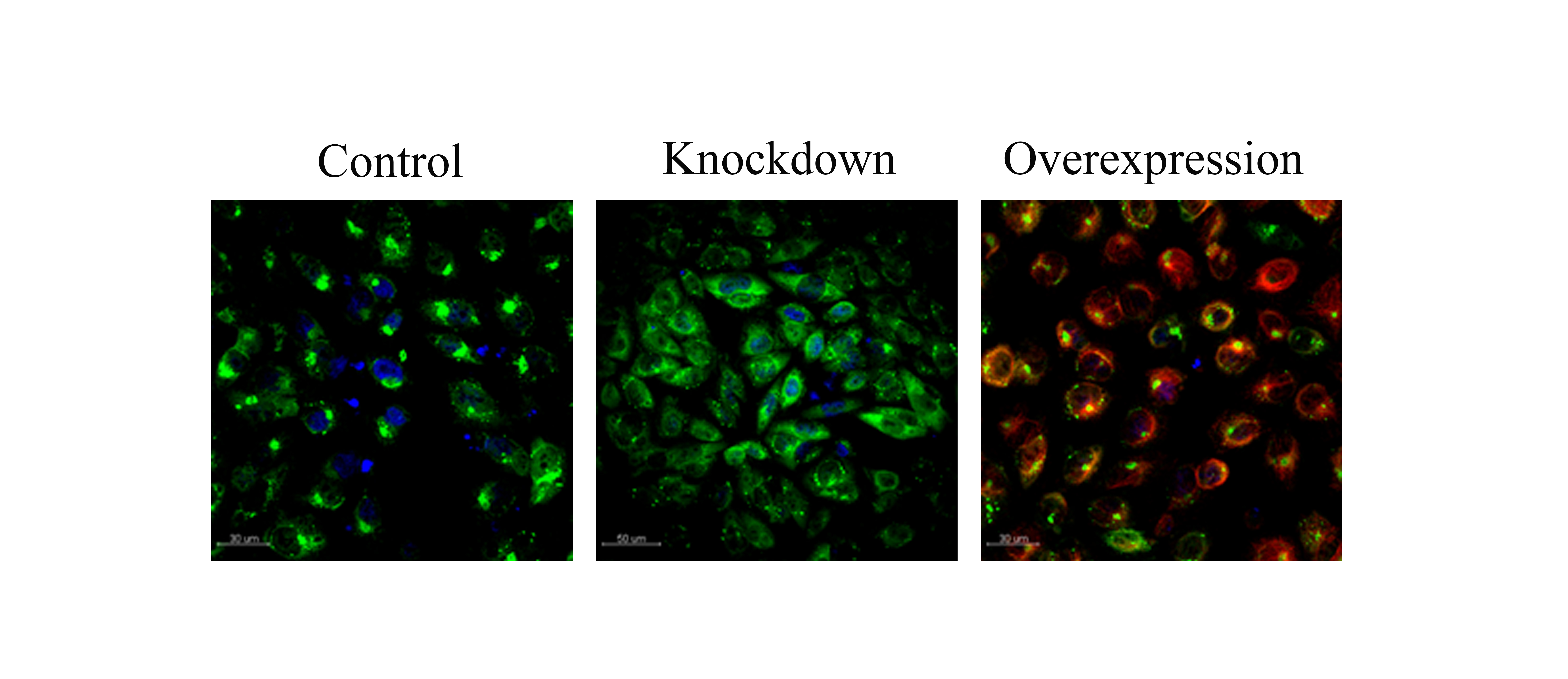

Supplement: Supplementary file 2 — Supplementary Fig 2 [file 41419_2019_1947_MOESM2_ESM.jpg]

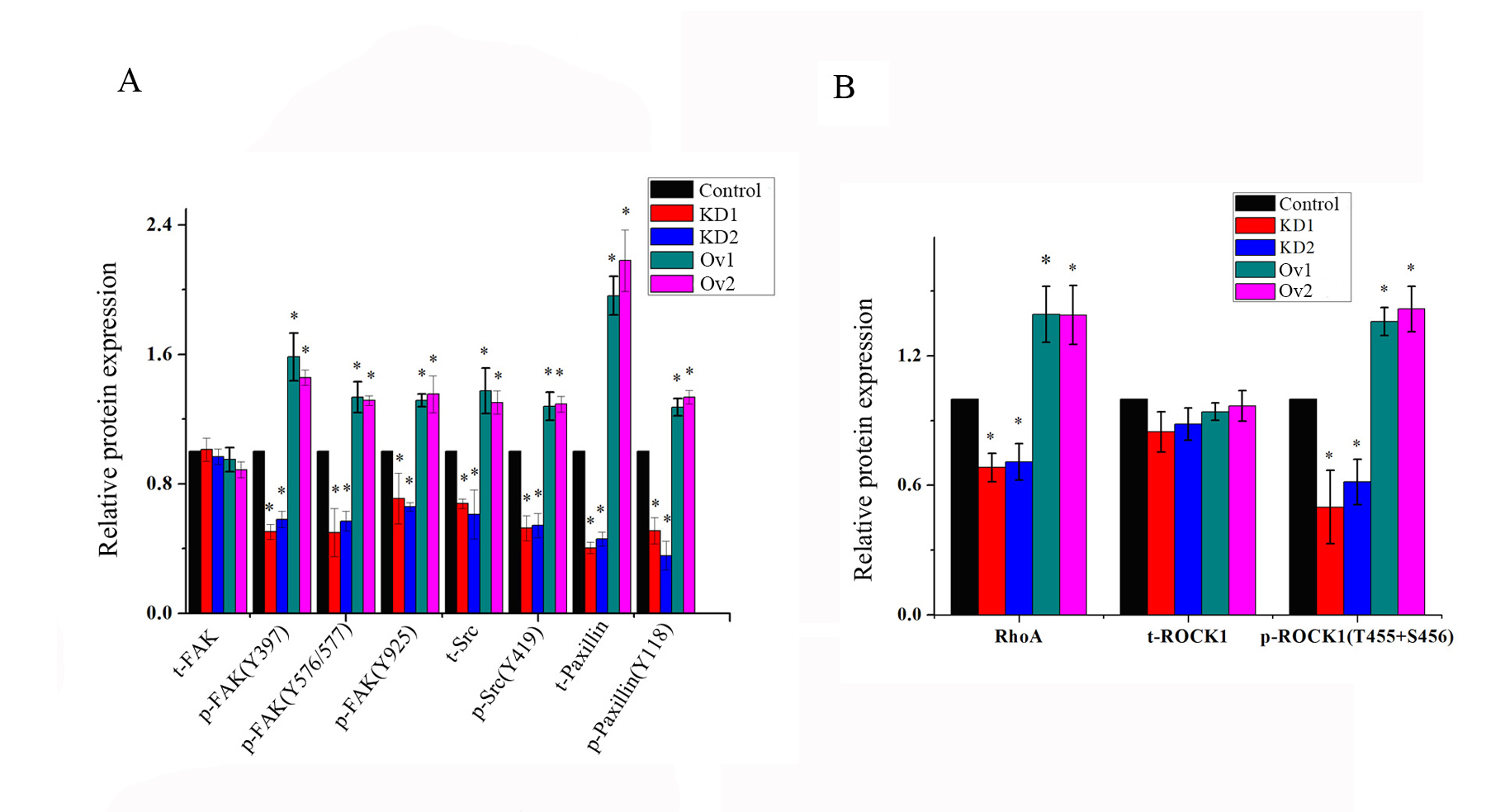

Supplement: Supplementary file 3 — Supplementary Fig 3 [file 41419_2019_1947_MOESM3_ESM.jpg]

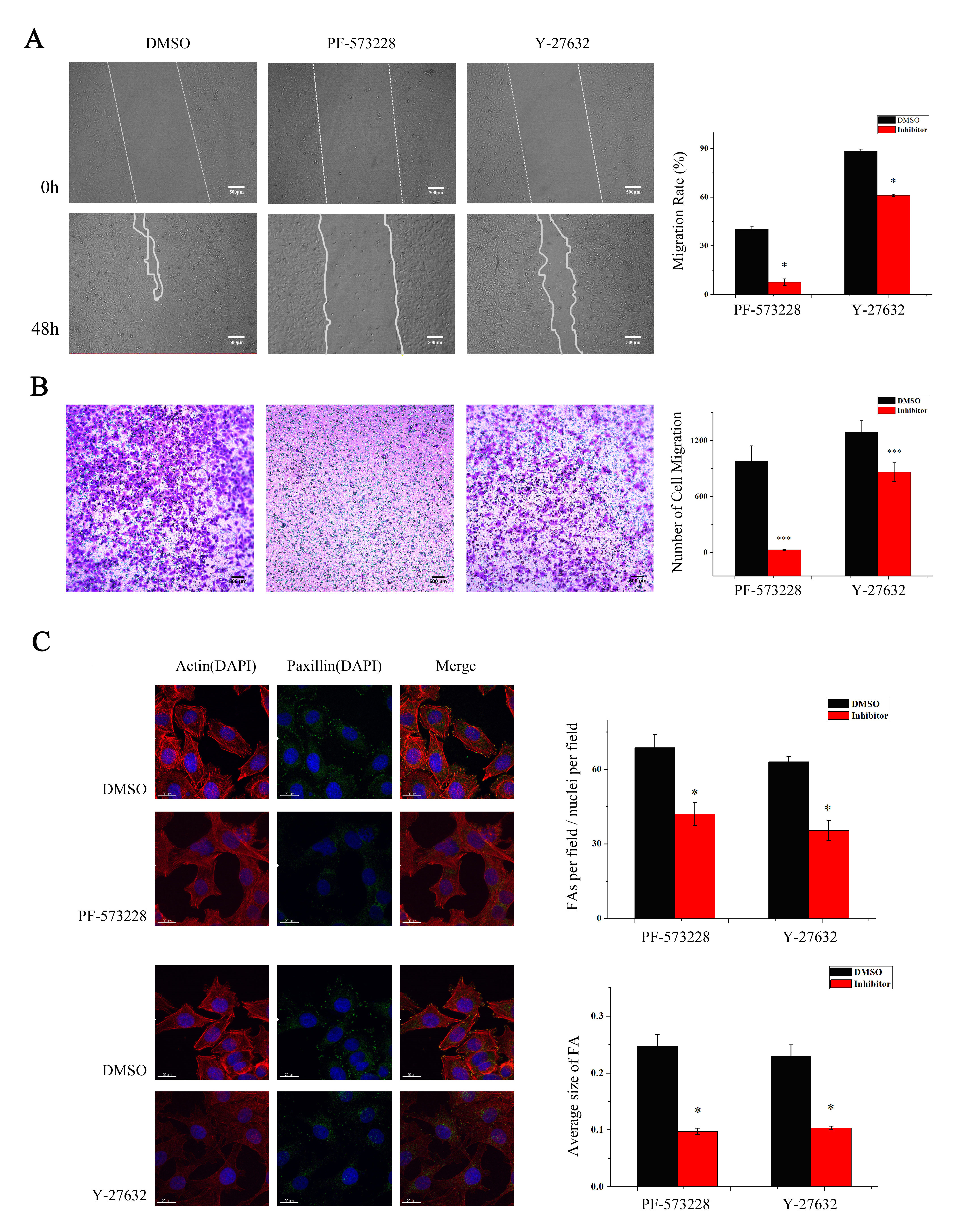

Supplement: Supplementary file 4 — Supplementary Fig 4 [file 41419_2019_1947_MOESM4_ESM.jpg]
